# Effect of herbal medicine Shenghua decoction on uterine bleeding after early medical abortion: A protocol for a systematic review and meta-analysis

**DOI:** 10.1097/MD.0000000000022944

**Published:** 2020-10-30

**Authors:** Ran Cheng, Shuhua Liu, Jianghong Gu, Lvyan Xu

**Affiliations:** Department of Obstetrics and Gynecology, Hangzhou Hospital of Traditional Chinese Medicine, Hangzhou, Zhejiang, China.

**Keywords:** medical abortion, meta-analysis, protocol, Shenghua decoction, systematic review

## Abstract

**Background:**

Excessive and prolonged uterine bleeding is an important obstacle for medical abortion to get popularized. Shenghua decoction (SHD) is widely used for treating uterine bleeding after early medical abortion. However, the clinical evidence is unclear.

**Methods:**

Two researchers will dependently search literatures of SHD for the treatment of uterine bleeding after medical abortion from Web of Science, PubMed, Embase, and The Cochrane Library; traditional Chinese medicine databases; China National Knowledge Infrastructure (CNKI); Chinese Scientific Journal Database (VIP database); and Wan-Fang Database. These inclusive data of included studies will be conducted by RevMan V5.3 software.

**Results:**

This systematic review and meta-analysis will provide a detailed summary of the current evidence related to the efficacy of SHD in treating uterine bleeding after early medical abortion, including the duration and volume of uterine bleeding, the medical abortion pain.

**Conclusion:**

This systematic review and meta-analysis will provide a detailed summary of the current evidence related to the efficacy of SHD in treating uterine bleeding after early medical abortion,

**Registration Number:**

PROSPERO CRD42020184465.

## 1 Introduction

### 1.1 Description of the condition

Unintended pregnancy is a problem that women encounter throughout their reproductive age. Around 1 in 4 women will experience an abortion in their lifetime, and about 55.7 million abortions take place worldwide every year.^[1]^ In China, over 9 million induced abortions were performed every year in recent years. Most induced abortions were performed in the first trimester.^[2]^ Induced abortion in the first trimester could be performed surgically (by manual or electric vacuum aspiration) or with medication. Though both methods have similar complete abortion rates, medical abortion is more natural and less harmful for its simplicity of operation.^[3]^ Medical termination of pregnancy has played a crucial role in providing access to safe, effective alternative worldwide by being noninvasive.^[4]^ The sequential combination of mifepristone with misoprostol is the regimen of choice for medical abortion nowadays.^[3,4–6]^

However, women who are eligible for medical abortion may still choose surgical abortion. In 2016, only 41.9% were completed by medical abortion among those that were eligible for early medical abortion on the basis of gestational age in America.^[7]^ Excessive and prolonged uterine bleeding is one of the most common and critical adverse reactions of medical abortion, for it increases the number of follow-up visits and risk of intrauterine infection.^[8]^ Therefore, reducing the uterine bleeding is very important to improve the satisfaction of medical abortion.

### 1.2 Description of the intervention

Chinese herbal medicine has been used to alleviate the postpartum hemorrhage in China for thousands years. According to traditional Chinese medicine (TCM) theory, “blood stasis” is the main etiology of postpartum hemorrhage, leading to pain in lower abdominal pain and uterine bleeding.^[9]^ Therefore, promoting blood circulation to dispel blood stasis is the primary method for reducing postpartum hemorrhage.

The TCM etiology of abnormal uterine bleeding induced by medical abortion is similar to postpartum hemorrhage. Shenghua decoction (SHD) is a very famous formula that has been used for treating postpartum hemorrhage in China since the Ming dynasty. SHD was created by Qingzhu Fu (A.D. 1606), an outstanding gynecologist in his times, and was described in his famous works “*Fu Qingzhu Nv Ke”.* It was made up of 5 kinds of herbal medicine: Angelicae Sinens Radix, Chuanxiong Rhizoma, Persicae Semen, Licorice, Common Ginger. The principle of formulating prescription of SHD was based on the blood stasis theory of TCM.

Nowadays, SHD is widely used for the treatment of uterine bleeding after medical abortion.^[10]^ It was reported that residual decidual tissues was the main reason for heavy and prolonged bleeding following the medical abortion.^[11,12]^ Experimental studies revealed that SHD could promote the discharge of decidual tissues by modulating the estradiol, estrogen receptor, progesterone receptor, bronectin, and laminin levels.^[13]^ Besides, uterine bleeding after medical abortion was demonstrated negative correlation with the proportions of Th1 and Th17 cells, and positive correlation with Th2 and Treg cells. SHD could efficiently induce the uterine bleeding by inducing Th1 and Th17 skews in the maternal–fetal of medical abortion patients.^[14]^

### 1.3 Why is performing this review important?

Medical abortion is safe and effective for termination of early pregnancy, however, the acceptability is not high as expected. Excessive and prolonged uterine bleeding is an important obstacle to get popularized. SHD is widely used for the treating uterine bleeding after medical abortion. Currently, no relevant systematic reviews of the efficacy of SHD for treating uterine bleeding after early medical abortion are conducted.

### 1.4 Objectives

To systematically evaluate the efficacy of SHD in treating the uterus bleeding after early medical abortion.

## 2 Methods /design

This study has been registered with international Prospective Register of Systematic Reviews (PROSPERO): CRD42020184465. The protocol has been drafted with the guidance of the Preferred Reporting Items for Systematic Reviews and Meta-Analysis Protocols (PRISMA-P).^[15]^

### 2.1 Criteria for including studies in this review

#### 2.1.1 Type of studies

Only randomized controlled trials (RCTs) will be included, without restrictions on language and publication status.

#### 2.1.2 Types of participants

This study will include women who performed early medical abortion at ≤63 days gestation with mifepristone and misoprostol, regardless of race, marital status or education, and economic status.

#### 2.1.3 Types of interventions and comparisons

Studies that used an SHD or a modified SHD will be included. SHD will include the following 5 formulas: Angelicae Sinens Radix, Rhizoma Chuanxiong, Semen Persicae, Licorice, Common Ginger. Modified SHD prescribe according to TCM syndrome differentiation will be acceptable and be defined by practitioners as adding only herbs to the original herbs, resulting in nearly the same actions as the original SHD. All types of herbal medicines will be included. There is no limitation on the number of herbs, administration methods dosage. SHD combined with any complementary therapy will be excluded, for example, acupuncture, moxibustion, and other complementary therapy. The control groups will consist of no treatment, placebo, and medication.

### 2.2 Types of outcome measures

#### 2.2.1 Primary outcomes

The duration and volume of uterine bleeding will be the primary outcomes. The duration was recorded from the first day of uterine bleeding to when the bleeding ceased completely. The unit of measurement was in days (d). The volume of uterine bleeding was measured by the weighing method, the alkaline hematin photometric method and other methods. The unit of measurement was in mL.

#### 2.2.2 Secondary outcomes

The medical abortion pain will be the secondary outcome. A reduction in pain that occurs only during the intervention or occurred as a result of the intervention, measure by a visual analogue scale (VAS), other validated scales, or as a dichotomous outcome.

### 2.3 Search methods in the study

The following databases will be searched by 2 independent review authors from September 2020 to November 2020: Web of Science, PubMed, Embase, and The Cochrane Library; Traditional Chinese Medicine databases; China National Knowledge Infrastructure (CNKI); Chinese Scientific Journal Database (VIP database); and Wan-Fang Database. Studies will also be hand-searched from the reference lists of all retrieved articles. Besides, we will search the trial registered platforms to obtain ongoing or unpublished trials. The trial registered platforms will be searched as follows: Clinicaltrials.gov (http://www.clinicaltrials.gov). A search strategy for PubMed has been established on the guidance of the Cochrane handbook guidelines:

#1 (“Medical abortion ” [Title/Abstract]) OR (“Induced Abortion ” [Title/Abstract])) OR (“Abortions, Induced ” [Title/Abstract])) OR (“Abortion (Induced) ” [Title/Abstract])) OR (“Abortion, Drug-Induced ” [Title/Abstract])) OR (“Abortion, Drug Induced ” [Title/Abstract])) OR (“Drug-Induced Abortion ” [Title/Abstract])

#2(“Shenghua decoction”[Title/Abstract]) OR(“Shenghua formula”[Title/Abstract]) OR (“Shenghua tang”[Title/Abstract])

#3 (“Randomized, controlled trial” [MeSH Terms]) OR (“Ran- domized controlled trial∗” [Title/Abstract]) (“clinical study” [Title/ Abstract]) OR (“Clinical Trial” [Title/Abstract]) OR (“Controlled study∗” [Title/Abstract]) OR (“Controlled Trial∗” [Title/Abstract])

#1 AND #2 AND #3

### 2.4 Data collection and analysis

#### 2.4.1 Selection of studies

**Figure 1:**
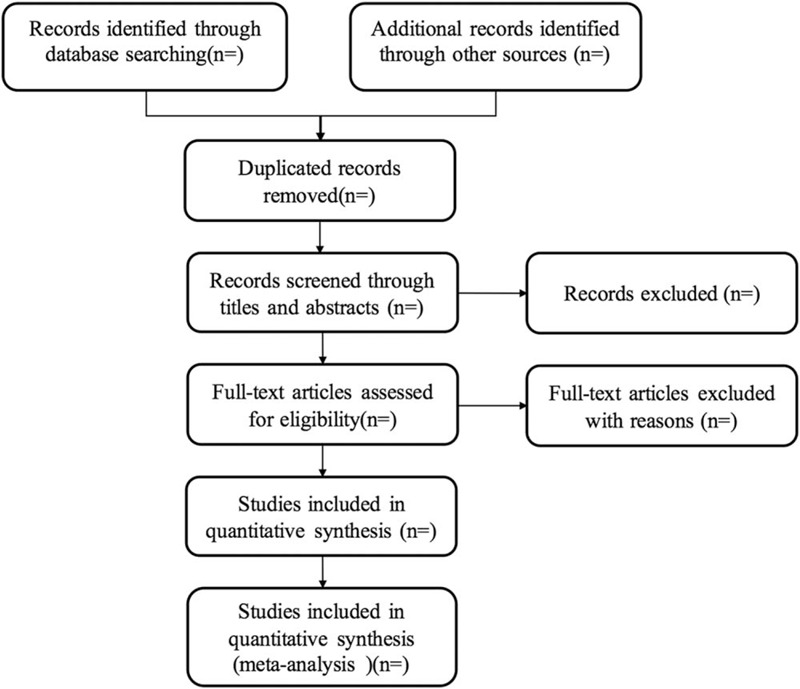
Study flow diagram.

Two reviewers will scan all retrieved research titles and abstracts independently to include eligible trials according to the predefined eligibility criteria. Full texts will be read when they have difficulty in identifying whether the study is included or not. All the eligible articles will be labeled as “included” or “excluded”, and the reasons of exclusion will be recorded. The selection process is shown in Figure 1. If it meets any disagreements, they will be solved by consulting the third review author.

#### 2.4.2 Data extraction and management

All the authors will design an extraction form, which including general information, methods, participants, interventions, outcomes, results, adverse events, and other information. Two reviewers will independently carry out the data extraction using predefined standard data collection form. In the process, any differences will be discussed by the 2 review authors, and any discrepancies will be resolved by consulting a third author. All extracted data will be inputted into RevMan V5.3 (Cochrane Community, London, UK) software for analysis.

#### 2.4.3 Assessment the risk of bias in studies

We will use Cochrane Handbook for Systematic Reviews of Interventions Tool as standard criteria for judging the risk of bias for each qualified trial. All included studies will be independently assessed by 2 authors. Disagreements will be discussed and arbitrated by the third review author. The assessment of risk and bias includes sequence generation, blinding of participants and personnel and outcome assessors, allocation sequence concealment, selective outcome reporting. It will be classified into 3 levels: “high risk”,“low risk”, or “unclear”.

#### 2.4.4 Data analysis

We will extract the data from parallel-group studies for analysis. The end of the treatment data or the end of the follow-up data will be extracted for assessment. In all studies, every single data for each outcome from every participant is collected and analysis.

#### 2.4.5 Missing data

We will contact original authors of the included studies to get missing or insufficient trial data by telephone or email. If the data still can not be obtained, we will just analyze the available data and discuss the potential impact of missing data.

### 2.5 Assessment of reporting biases

Funnel plots will be adopted for assessing the reporting biases and small-study effects for all eligible trials, regardless of their methodological quality. The risk and bias assessment includes sequence generation, personnel and outcome assessors, selective outcome reporting, incomplete outcome data, and other sources of bias. If 10 or more trials studies are included in the study, a test for funnel plot asymmetry using Egger method will be conducted.^[26]^

### 2.6 Data synthesis

RevMan V5.3 statistical software will be used for data synthesis. We will use mean difference or standardized mean difference and 95% confidence intervals (CIs) for continuous data. We will use the RR with 95% confidence interval (CI) for dichotomous outcomes. *I*^2^ test will be utilized to evaluate the heterogeneity. If no significant heterogeneity exists (*I*^2^ < 50), the fixed-effects model will be conducted. Otherwise, the random-effects model will be used (*I*^2^ ≥ 50). If quantitative synthesis is not appropriate, we will adopt subgroup analysis to describe the characteristics of the included trials.

### 2.7 Subgroup analysis

We do not have pre-subgroup plan in our review. If there are significant heterogeneity exists, subgroup analysis will also be applied possibly under certain circumstances.

### 2.8 Sensitivity analysis

A sensitivity analysis will be performed to assess the robustness of the meta-analysis results if needed. Sensitivity analysis is used to analyze the quality of research, methodological elements, type of publication etc. If there is a high degree of heterogeneity, we will exclude low-quality or small sample studies to repeat the meta-analysis. If the results are changed, more caution should be taken when drawing conclusions.

### 2.9 Grading the quality of evidence

The Grading of Recommendations Assessment, Development and Evaluation (GRADE) will be utilized to evaluate the evidence quality for the main outcomes. We will assess the quality of evidence into 4 levels: high, moderate, low, and very low according to GRADEs classification.

## 3 Discussion

Nowadays, SHD is widely used throughout China and elsewhere in the world for the treatment of uterine bleeding after early medical abortion. To our best knowledge, no systematic review has specifically addressed this issue. Our systematic review will provide a detailed summary of the current evidence related to the efficacy of SHD in treating uterine bleeding after early medical abortion. This evidence will be useful to practitioners, patients, and health policy-makers regarding the use of SHD after early medical abortion.

## Author contributions

RC conceived and designed the study. RC developed the search strategy. SHL, JHG, YLX, and RC wrote and prepared the protocol. JHG, YLX, and SHL planned the data extraction and aimed to perform the analysis. SHL provided critical comments. All authors read and critically revised the protocol.

**Conceptualization:** Jianghong Gu, Lvyan Xu.

**Data curation:** RAN Cheng, Shuhua Liu, Lvyan Xu.

**Formal analysis:** Shuhua Liu.

**Methodology:** RAN Cheng, Jianghong Gu, Lvyan Xu.

**Project administration:** Lvyan Xu.

**Supervision:** RAN Cheng.

**Validation:** RAN Cheng, Lvyan Xu.

**Writing – original draft:** RAN Cheng, Jianghong Gu.

**Writing – review & editing:** RAN Cheng, Shuhua Liu, Lvyan Xu.
